# Effect of the ratio of dietary metabolizable energy to nitrogen content on production performance, serum metabolites, rumen fermentation parameters, and bacterial diversity in yaks

**DOI:** 10.3389/fmicb.2022.1013980

**Published:** 2022-10-11

**Authors:** Xiaojing Liu, Jie Li, Lizhuang Hao, Allan Degen, Dongyang Wang, Yonggui Ma, Jianzhang Niu, Yanfen Cheng, Shujie Liu

**Affiliations:** ^1^Laboratory of Gastrointestinal Microbiology, National Center for International Research on Animal Gut Nutrition, Nanjing Agricultural University, Nanjing, China; ^2^Key Laboratory of Plateau Grazing Animal Nutrition and Feed Science of Qinghai Province, Qinghai Academy of Animal Science and Veterinary Medicine of Qinghai University, Xining, China; ^3^Gansu Polytechnic College of Animal Husbandry & Engineering, Wuwei, China; ^4^Desert Animal Adaptations and Husbandry, Wyler Department of Dryland Agriculture, Blaustein Institutes for Desert Research, Ben-Gurion University of the Negev, Beer Sheva, Israel; ^5^Academy of Plateau Science and Sustainability, People’s Government of Qinghai Province and Beijing Normal University, Key Laboratory of Medicinal Animal and Plant Resources of Qinghai-Tibetan Plateau in Qinghai Province, College of Life Science, Qinghai Normal University, Xining, China; ^6^State Key Laboratory of Grassland Agro-Ecosystems, Center for Grassland Microbiome, College of Pastoral Agriculture Science and Technology, Lanzhou University, Lanzhou, China

**Keywords:** yak, ratio of metabolizable energy to nitrogen, average daily gain, blood biochemistry, rumen fermentation, rumen bacteria

## Abstract

This study examined the effect of the ratio of dietary metabolizable energy (MJ) to nitrogen (g) content (ME:N) on average daily gain (ADG), blood biochemical indices, rumen fermentation parameters, and rumen bacterial community in yaks. Thirty-six male yaks, aged 2–3 years, were divided into three groups and received a ME:N ratio of 0.42 (HY), 0.36 (MY,) or 0.32 (LY) MJ/g. Dry matter intake ranged between 3.16 and 3.63 kg/d and was lesser (*p* < 0.001) in the LY group than the other two groups. ME intake increased (*p* < 0.001) with an increase in the ME:N ratio, while N intake did not differ among groups. The ADG was 660 g/day for the MY group, which was higher (*p* < 0.005) than the 430 g/day in the LY group, while the HY group gained 560 g/day and did not differ from the other two groups. Feed intake to ADG ratio ranged between 5.95 and 7.95, and numerically was highest in the LY group and lowest in the MY group. In general, the concentration of ruminal total volatile fatty acids (*p* < 0.03) and molar proportions of propionate (*p* < 0.04), increased, while the molar proportion of acetate (*p* < 0.005) and the acetate:propionate ratio decreased (*p* < 0.001) with a decrease in the ME:N ratio. The molar proportion of butyrate did not differ among groups (*p* = 0.112). Group MY had higher ruminal NH_3_-N content than group HY and had a higher serum glucose content but lower urea content, lactate dehydrogenase, and creatine kinase content than group LY. In ruminal bacteria at the phylum level, the relative abundance of Firmicutes (F) was greater and of Bacteroidetes (B) was lesser, while the F:B ratio was greater in group MY than in groups HY an LY. We concluded that the yaks consuming the diet containing a ME:N ratio of 0.36 MJ/g had the best performance of the three groups.

## Introduction

Yaks are multifunctional and dominant livestock on the Qinghai-Tibetan plateau (QTP), providing meat, milk, fiber, dung, and transportation (Long et al., 2008). They are vital to the livelihood and play important roles in the cultural and religious traditions of local residents (Joshi et al., 2020). Yaks have adapted well to the harsh climate of the QTP, which is characterized by severe environmental conditions, namely low air temperature and oxygen concentration, high ultraviolet light, and a short growing season of forage (Miao et al., 2015). Traditionally, yaks graze in natural pasture all year round without supplements. Much of the grassland is degraded and forage is sparse and of poor quality in the winter, resulting in considerable body weight loss in the yaks. With the drive for ecological protection and restoring the degraded grassland, yak production is gradually introducing feedlot fattening.

Dietary energy and protein are important factors affecting the digestion and metabolism of nutrients in livestock production (Schroeder and Titgemeyer, 2008). Protein and energy levels are closely related to nutrient degradation, rumen fermentation, and intestinal microbial structure. Appropriate energy-to-nitrogen (ME:N) ratio can improve energy and N utilization efficiency and reduce N losses in urine and feces (Marini and Amburgh, 2001; Yan et al., 2006; Hossein-Zadeh and Ardalan, 2011). Furthermore, it has been reported that the ratio can affect average daily gain (ADG), rumen fermentation parameters, and bacteria diversity in ruminants (Gabler and Heinrichs, 2003).

With the increase in feedlot fattening of yaks, more information is needed on their energy and protein requirements. Often, requirements are adapted from cattle, but these two bovine species are different in their requirements. Moreover, the research on yaks has focused mainly on the effects of different energy levels on their metabolism and rumen microbial diversity (Ahmad et al., 2020; Hu et al., 2020), and more information is needed on optimal dietary energy and protein levels. Therefore, in this study, we offered yaks different ratios of ME:N and determined their dry matter intake (DMI), average daily gain (ADG), blood biochemistry, rumen fermentation parameters, and the diversity and structure of rumen bacteria. The results could provide a basis for optimal ME:N ratios for feedlot-raised yaks.

## Materials and methods

The study was conducted at the Animal Nutrition Laboratory, Academy of Animal Husbandry and Veterinary Sciences, Qinghai University, Xining, Qinghai Province, China (101.52°E, 37.4°N, 2998 m above sea level). The site has a plateau continental climate. The protocol and all experimental procedures on the animals were approved by the Committee of Animal Use, Academy of Science and Veterinary Medicine, Qinghai University (Protocol QMKY202002).

### Animal feeding and sampling

Thirty-six 2–3 year old male yaks were divided randomly into three groups based on the dietary metabolizable energy:nitrogen content (ME:N), namely 0.42 MJ/g (HY), 0.36 MJ/g (MY), and 0.32 MJ/g (LY). The dietary rations were prepared based on the NRC beef cattle feeding standard and relevant research on the nutritional needs of yaks (Han et al., 1993; Xue et al., 1994). Dietary composition and nutritional level of each diet are presented in Table 1.

**Table 1:** Dietary components and chemical composition of diets with different ME:N ratios (DM basis).

Items	Groups		
	High ME:N (0.42 MJ/g)	Medium ME:N (0.36 MJ/g)	Low ME:N (0.32 MJ/g)
Raw material composition			
Oat green hay	7.5	10	15
Corn silage	30	20	10
Corn kernels	40.5	40	30
Bran	10	17	20
Soybean meal	5	5	10
Rapeseed cake	5	5	9
Mineral-vitamin premix	2	2	2
Baking soda	0	1	1
Expanded Urea	0	0	0.5
Stone powder	0	0	1.5
Salt	0	0	1
Chemical Composition			
DM (g/kg)	637.3	608.9	706.5
EE (g/kg)	27.6	27.7	27.2
CP (g/kg)	153.9	161.9	176.0
NDF (g/kg)	542.2	477.8	481.2
ADF (g/kg)	161.1	186.7	172.0
Gross energy (MJ/kg)	16.4	15.1	14.6
ME (MJ/kg)	10.4	9.4	9.1
ME:N ratio (MJ/g)	0.42	0.36	0.32

The yaks were kept in an open-sided roofed structure. They were penned (1.5 m × 3 m) individually, which allowed measurement of individual feed intake, and were fed *ad libitum* at 08:00 and 17:00 daily, with water freely available. The diet was offered for 28 days, and on day 29, before morning feed, the yaks were weighed, and jugular vein blood and rumen fluid were collected. Blood samples were collected by vacuum tubes, centrifuged at 3000 × g for 20 min and serum samples were stored at −20°C. Approximately 300 ml of rumen fluid was collected using a flexible oral stomach tube with a metal strainer (Anscitech Co. Ltd., Wuhan, China). The first 200 ml of fluid was discarded to eliminate saliva contamination, and the equipment was washed thoroughly with clean warm water between collections. The rumen fluid was filtered through four layers of gauze, pH was measured with a pH meter (EcoScan pH 5, Singapore), and then, the rumen fluid was stored at liquid nitrogen tank for determination of fermentation parameters and microbial extraction.

### Determination of the chemical composition of the diets, blood biochemical indices, and rumen fermentation parameters

Feed samples of the three treatment diets were collected in two consecutive days each week (*n* = 8 for each diet), and stored at −20°C. The samples were dried in an oven at 65°C for 48 h, passed through a 2 mm sieve, and then dried at 105°C for 8 h to determine dry matter content. Gross energy of the diets was measured by an oxygen bomb calorimeter (German IKA C6000 gs, Staufen, Germany), and EE by Soxhlet extraction using petroleum ether (AOAC, 2010). Total N was determined by the Kjeldahl method and crude protein was calculated as N × 6.25. Neutral detergent fiber (NDF) concentration was assayed with heat stable α-amylase and sodium sulfite, and acid detergent fiber (ADF) concentration was determined using fiber bags and a fiber analyzer (ANKOM 200ifiber analyzer; ANKOM Technologies, Inc., Fairport, NY, United States) following Van Soest et al. (1991).

Serum biochemical determination kits (Nanjing Jiancheng Bioengineering Institute, Nanjing, China) were used to determine the concentrations of serum glucose (Glucose Assay Kit, F006-1-1), triglycerides (Triglyceride Assay Kit, A110-1-1), alkaline phosphatase (Alkaline Phosphatase Assay Kit, A059-2-2), total protein (Total Protein Quantitative Assay Kit, A045-2-2), albumin (Albumin Assay Kit, A028-2-1), urea (Urea Assay Kit, C013-2-1), creatine kinase (Creatine Kinase Assay Kit, A032-1-1), cholesterol (Total Cholesterol Assay Kit, A111-1-1), aspartate aminotransferase (Aspartate Aminotransferase Assay Kit, C010-2-1), alanine aminotransferase (Alanine Aminotransferase Assay Kit, C009-2-1), and lactate dehydrogenase (Lactate Dehydrogenase Assay Kit, A020-2-2). The colorimetric method was used for the detection of indicators. The concentration of globulin was taken as the difference between total protein and albumin (Jiang et al., 2015).

The concentration of ruminal NH_3_-N was determined by the colorimetric method (Shen et al., 2017), and of microbial crude protein by Coomassie brilliant blue method (Makkar et al., 1982). The concentrations of volatile fatty acids, acetate, propionate, isobutyrate, butyrate, isovalerate, and valerate were determined by gas chromatography (Daojin GC2014AFsc instrument, Shimadzu, Japan) at 40°C column temperature, 220°C injection temperature, and 230°C TCD temperature as detailed by Wang et al. (2011). The pressures of air, N_2_ carrier gas, and H_2_ were 0.05 Mpa. The standard curves were established and crotonic acid was used as the internal standard to calculate the concentration of the measured SCFA.

### Microbiome sequencing and bioinformatic analysis

Total DNA from rumen fluid was extracted using Power DNA Isolation Kit (Mo Bio Laboratories, Carlsbad, CA). The DNA quality and concentration were checked by NanoDrop (Thermo Fisher Scientific, Waltham, MA, United States), and the V3-V4 region of 16S rRNA was amplified by PCR. High-throughput sequencing primers were 338-F (5″- ACTCCTACGGGAGGCAGCAG-3″) and 806-R (5”-GGACTACCVGGGTATCTAAT-3″; Klindworth et al., 2013). Agencourt AMPure XP magnetic beads (Beckman Coulter, Milan, Italy) amplified the product into purification, library quality inspection was carried out, and qualified libraries were sequenced using Illumina Miseq platform. After obtaining the original results, cutadapt v2.6 software removed the primer sequence and barcode (Bolger et al., 2014), and QIIME2 was used for quality filtering, double-end stitching and chimera removal (Caporaso et al., 2010). Operational taxonomic units (OTUs) were clustered with a 99% similarity cutoff standard into different characteristic sequences using the Vsearch plug-in unit in QIIME2. The relative abundances of bacteria at the phylum and genus levels were obtained by species annotation using Silva 16S rRNA database SILVA_128. Alpha diversity and Beta diversity were calculated with QIIME 2 software. The principal coordinate analysis (PCoA) was used to compare the bacteria communities among groups based on the unweighted UniFrac distance (Lozupone et al., 2011). The common and special OTUs among groups were analyzed using the Venn diagram. The linear discriminant analysis (LDA) effect size (LEfSe) was used to detect the dominant bacterial community differences among groups (Segata et al., 2011). To predict taxa functions, the Kyoto Encyclopedia of Genes and Genomes (KEGG) pathway hierarchy level 3 was applied using PICRUSt software (Langille et al., 2013). A Spearman’s correlation heatmap analyzed the correlations between the different phenotypic indices and bacteria. Afterward, the raw sequence data were subjected to the Sequence Read Archive (SRA) of the NCBI under accession number PRJNA835682.

### Data processing and analysis

The ME of the three diets was calculated as: DE = GE × [91.6694–91.3359 × (ADF-OM)]; ME = DE*0.82 (Chinese feeding standard NY/T815-2004). One-way ANOVA and LSD for multiple comparisons tested for differences among dietary treatments in dry matter intake (DMI), ADG, blood biochemical indices, and rumen fermentation parameters (SPSS 26.0, Chicago, IL, United States). A level of *p* < 0.05 was accepted as significant, and results are expressed as mean ± standard error of mean (SEM). Stacked histograms were plotted by Graph Prism v6.^1^ Bar and box graphs, PCoA, the Venn diagram, LEfSe, and heatmap graphs were plotted using the online website,^2^,^3^ in which the LDA threshold of comparative analysis of rumen bacterial abundance was greater than 3, and of bacterial differential function was greater than 2.

## Results

### Dry matter intake and average daily gain

The DMI ranged between 3.16 and 3.63 kg/d and was lesser in the LY group than the other two groups (*p* < 0.01). The ME intake increased (*p* < 0.001) with an increase in the ME:N ratio, while there was no difference among groups in N intake (Table 2). The ADG was 660 g/day for group MY, which was greater (*p* < 0.005) than the 430 g/day in group LY, while group HY gained 560 g/day and did not differ from the other two groups. Feed intake to ADG ratio ranged between 5.95 and 7.95, and numerically was the highest in group LY and the lowest in group MY.

**Table 2:** Effect of dietary ME:N ratio on dry matter intake, average daily gain, and feed:gain ratio in yaks.

Items	Groups		SEM		*P*-value
	High ME:N (0.42 MJ/g)	Medium ME:N (0.36 MJ/g)	Low ME:N (0.32 MJ/g)		
BW (kg)	161.8	163.2	163.0	2.36	0.968
DMI (kg/d)	3.63^a^	3.51^a^	3.16^b^	0.06	0.005
ADG (g/d)	560^ab^	660^a^	430^b^	30.00	0.005
F:G (kg/kg)	6.66	5.95	7.95	0.39	0.100
MEI (MJ/d)	37.9^a^	32.9^b^	28.6^c^	0.84	<0.001
NI (g/d)	89.5	90.9	88.9	3.00	0.855

BW, Initial body weight; DMI, Dry matter intake; ADG, Average daily gain; F:G, Feed to gain ratio; MEI, ME intake; and NI, N intake. a-c Different superscripts in the same row indicate significant differences (*p* ≤ 0.05).

### Rumen fermentation parameters

Rumen pH in HY yaks was higher than in the MY and LY yaks (*p* < 0.001). Concentration of ruminal NH_3_-N decreased (*p* < 0.001) with an increase in the ME:N ratio, while microbial crude protein followed the same trend numerically, albeit the differences were not significant. In general, the concentrations of ruminal total volatile fatty acids (TVFAs; *p* < 0.03) and molar proportions of propionate (*p* < 0.04) increased, while the molar proportion of acetate (*p* < 0.005) and the acetate:propionate ratio decreased (*p* < 0.001) with a decrease in the ME:N ratio. The molar proportion of butyrate did not differ among groups (*p* = 0.112; Table 3).

**Table 3:** Effect of dietary ME:N ratio on rumen fermentation parameters in yaks.

Items	Groups			SEM	*P*-value
	High ME:N (0.42 MJ/g)	Medium ME:N (0.36 MJ/g)	Low ME:N (0.32 MJ/g)		
pH	6.53^a^	6.24^b^	6.18^b^	0.04	<0.001
NH_3_-N (mg/dL)	11.5^c^	15.4^b^	20.9^a^	0.73	<0.001
Microbial crude protein (mg/mL)	1.34	1.44	1.48	0.04	0.348
Acetate (mol/100 mol)	63.9^a^	60.9^a^	56.9^b^	0.92	0.004
Propionate (mol/100 mol)	19.2^b^	21.3^a^	22.0^a^	0.47	0.032
Isobutyrate (mol/100 mol)	2.00	1.79	2.02	0.16	0.811
Butyrate (mol/100 mol)	11.19	12.80	15.92	0.94	0.112
Isovalerate (mol/100 mol)	2.71	2.39	2.50	0.15	0.680
Valerate (mol/100 mol)	1.04^a^	0.80^ab^	0.73^b^	0.06	0.082
TVFA (mmol/L)	68.4^b^	82.2^ab^	90.2^a^	3.36	0.022
Acetate:Propionate	3.36^a^	2.93^b^	2.60^b^	0.09	<0.001

a-c Different superscripts in the same row indicate significant differences(*p* ≤ 0.05).

### Blood biochemical indices

The concentration of serum glucose in group MY was greater (*p* < 0.02) than in groups LY and HY; total protein in group MY and group LY was greater (*p* < 0.04) than in group HY; and, urea, lactate dehydrogenase, and creatine kinase in group LY were greater (*p* < 0.05) than in group HY and MY (Table 4). Concentrations of serum triglycerides, cholesterol, alkaline phosphatase, aspartate transaminase, alanine aminotransferase, albumin, and globulin did not differ among groups.

**Table 4:** Effect of dietary ME:N ratio on blood biochemical indices in yaks.

Items	Groups			SEM	*P*-value
	High ME:N (0.42 MJ/g)	Medium ME:N (0.36 MJ/g)	Low ME:N (0.32 MJ/g)		
Glucose (mmol/L)	4.49^b^	4.95^a^	4.43^b^	0.08	0.015
Triglyceride (mmol/L)	0.23	0.21	0.16	0.02	0.385
Alkaline phosphatase (U/L)	203	210	176	10.5	0.396
Total protein (g/L)	76.7^b^	82.2^a^	82.1^a^	1.01	0.032
Albumin (g/L)	35.0	37.1	36.8	0.44	0.096
Urea (mmol/L)	5.82^b^	5.55^b^	8.53^a^	0.40	0.001
Creatine kinase (U/L)	283^b^	284^b^	363^a^	12.2	0.006
Cholesterol (mmol/L)	1.77	1.88	1.76	0.06	0.656
Aspartate transaminase (U/L)	85.8	84.7	107.2	5.53	0.167
Alanine aminotransferase (U/L)	30.7	33.8	33.3	1.00	0.410
Lactate dehydrogenase (IU/L)	918^b^	911^b^	976^a^	10.96	0.026
Globulin (g/L)	41.7	45.1	45.3	0.78	0.107
Albumin: Globulin	0.84	0.83	0.82	0.04	0.842

a-c Different superscripts in the same row indicate significant differences(*p* ≤ 0.05).

### Ruminal bacteria

**Figure 1:**
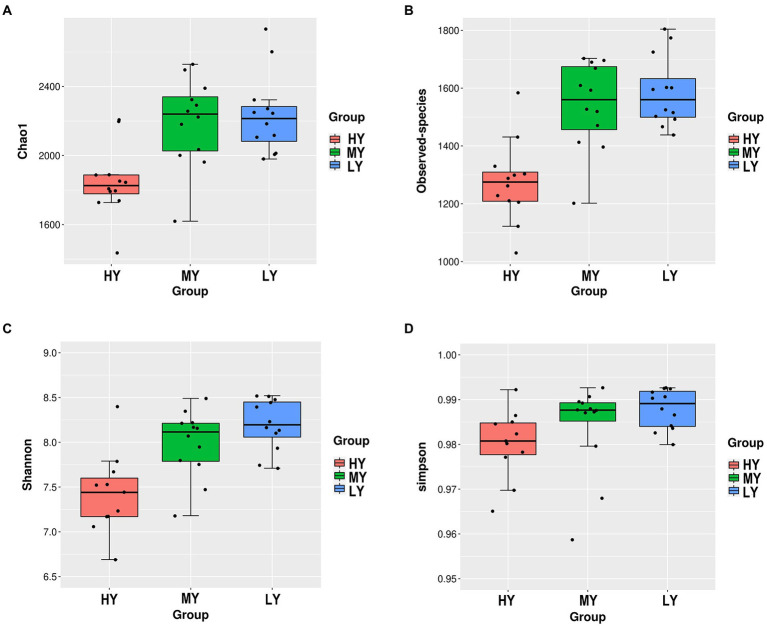
Differences in ruminal bacterial diversity and richness among groups. Bacterial richness was estimated by the observed-species and the Chao1 indices **(A,B)**. Bacterial diversity was estimated by Shannon and Simpson indices **(C,D)**. HY, high ME:N (0.42 MJ/g); MY, medium ME:N (0.36 MJ/g); LY, low ME:N (0.32 MJ/g).

For the α diversity indices (Table 5; Figure 1), observed-species, Chao1 and Shannon were the lowest (*p* < 0.001) in group HY, but the Simpson index did not differ among groups.

**Table 5:** Changes of different ME:N ratios on alpha diversity indices of rumen bacteria in yaks.

Items	Groups			SEM	*P*-value
	High ME:N (0.42 MJ/g)	Medium ME:N (0.36 MJ/g)	Low ME:N (0.32 MJ/g)		
Observed-species	1274^b^	1541^a^	1587^a^	94.5	<0.001
Chao1	1848^b^	2192^a^	2236^a^	56.7	<0.001
Shannon	7.29^b^	7.99^a^	8.20^a^	0.18	<0.001
Simpson	0.97	0.99	0.99	0.01	0.136

a-c Different superscripts in the same row indicate significant differences(*p* ≤ 0.05).

**Figure 2:**
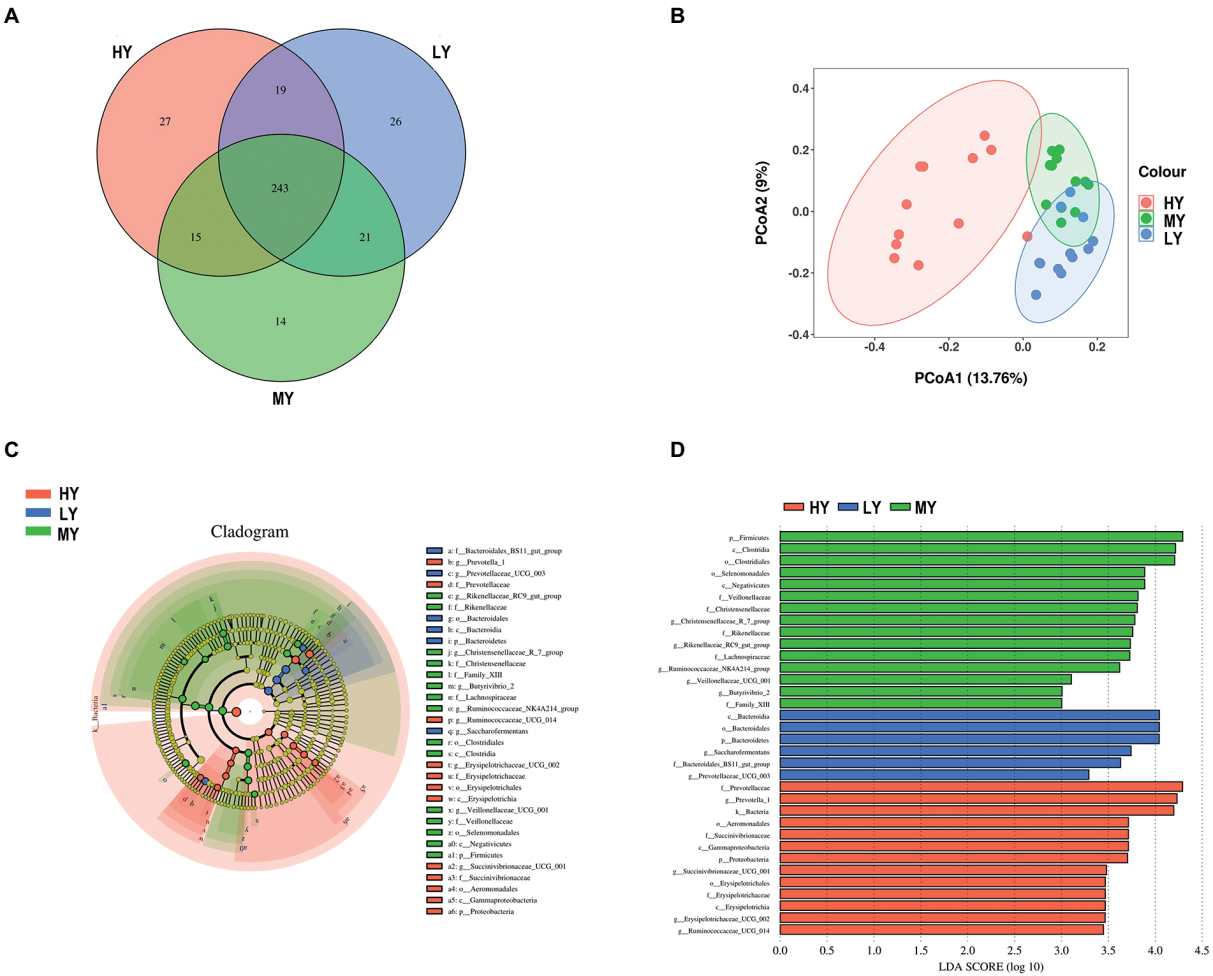
Venn diagrams **(A)** and PCoA plots **(B)** of the rumen bacterial community for the different ME:N ratios. LEfSe analysis of the bacterial community for the different ME:N. Cladogram **(C)** showing significantly enriched bacterial taxa from phylum to genus level. In the histogram **(D)**, the red color illustrates that the abundance of taxa was highest in the high ME:N (0.42 MJ/g); the green color illustrates that the abundance of taxa was highest in the medium ME:N (0.36 MJ/g); and the blue color illustrates that the abundance of taxa was highest in the low ME:N (0.32 MJ/g). HY, high ME:N (0.42 MJ/g); MY, medium ME:N (0.36 MJ/g); LY, low ME:N (0.32 MJ/g).

Based on Venn diagrams, there were 243 common taxa of bacteria, and 27, 14, and 26 unique taxa for the HY, MY, and LY groups, respectively (Figure 2A). There were also significant differences in rumen bacterial community structure and diversity among groups. For beta diversity, PCoA analysis revealed that the bacterial community structure of rumen fluid had clear structural separation among groups based on the Bray-Curtis index (Figure 2B). Differential bacteria were identified from the kingdom to genus levels using the LEfSe analysis. By setting the LDA value to 3 and analyzing the difference of rumen bacterial communities among groups, the rumen bacterial community responded significantly to the ME:N ratio (Figures 2C,D).

**Figure 3:**
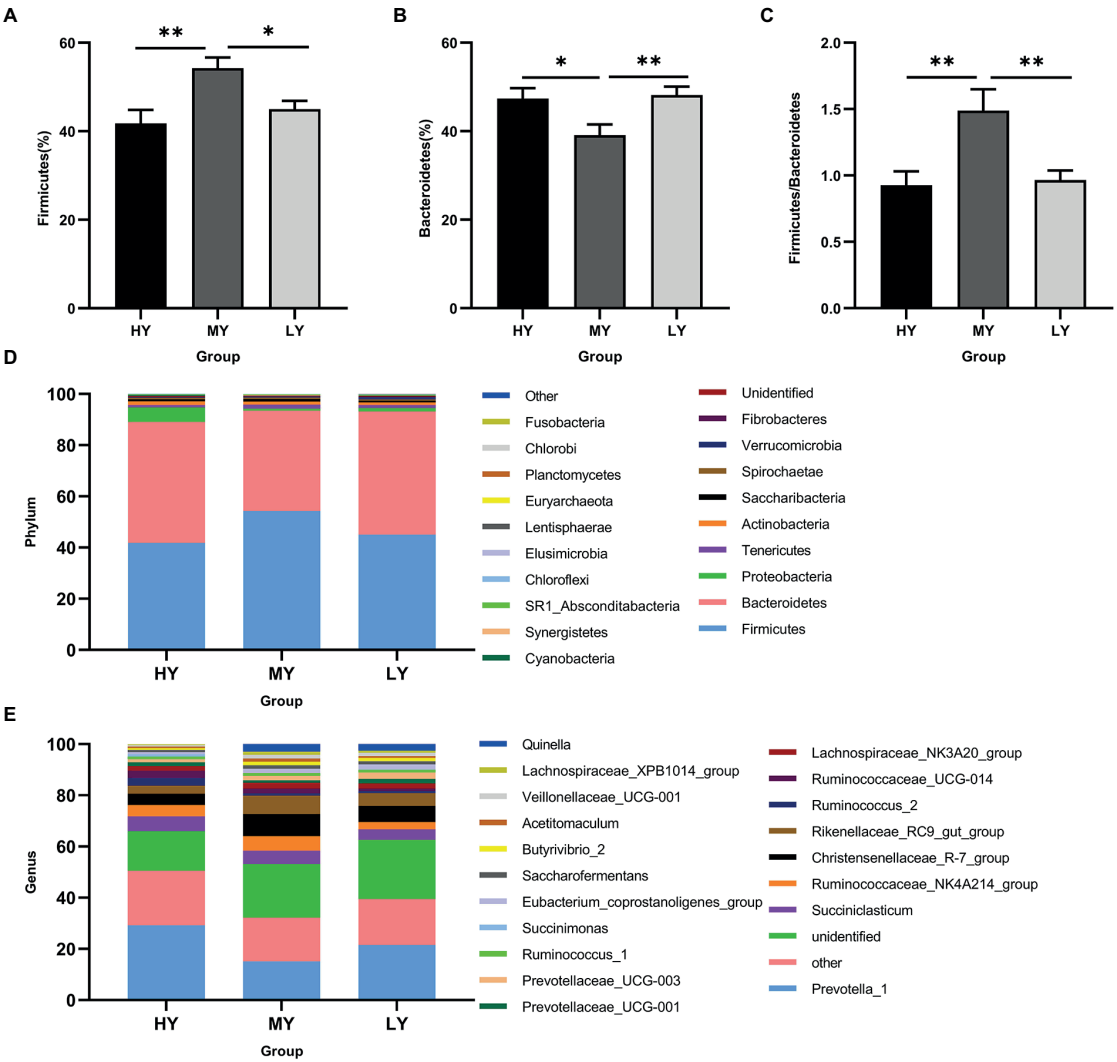
Changes in the relative abundance of the dominant phylum and genera in the rumen bacteria among of yak groups. These are **(A)** Firmicutes, **(B)** Bacteroidetes, and **(C)** the ratio of Firmicutes to Bacteroidetes. Relative abundances of bacteria at phylum level **(D)** and genus level **(E)** for different ME:N ratios. HY, high ME:N (0.42 MJ/g); MY, medium ME:N (0.36 MJ/g); LY, low ME:N(0.32 MJ/g). ^*^p < 0.05 and ^**^p < 0.01.

**Figure 4:**
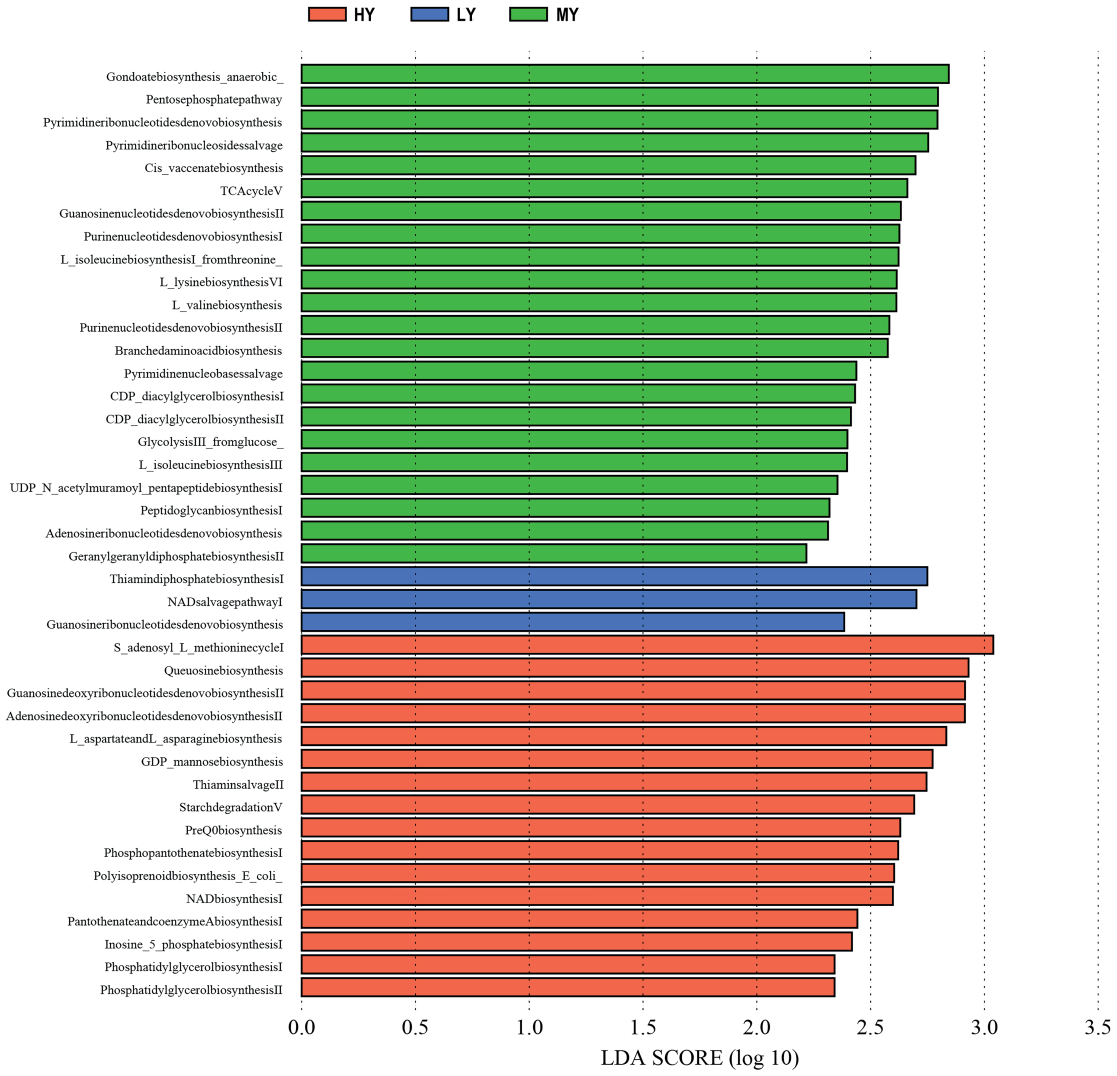
The correlation heat maps between the production performance index, rumen fermentation, blood biochemical indices, and significant different bacteria.

**Figure 5:**
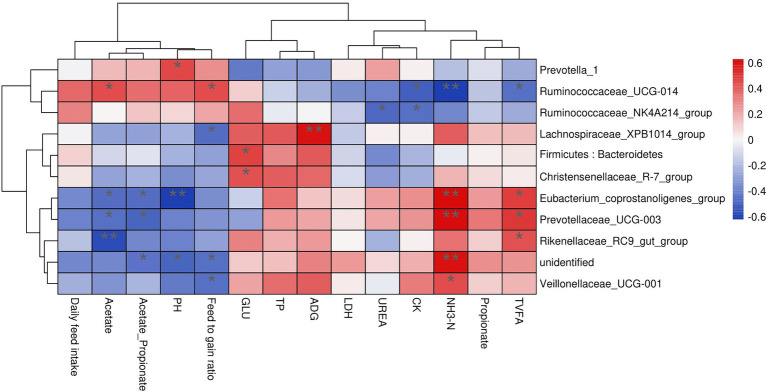
Analysis of LEfSe difference in KEGG metabolic pathway of rumen bacteria. HY, high ME:N (0.42 MJ/g); MY, medium ME:N (0.36 MJ/g); LY, low ME:N (0.32 MJ/g).

Rumen bacteria were dominated by the phyla Firmicutes, Bacteroidetes, Proteobacteria, and Tenericutes (Figures 3A–D). The relative abundance of Firmicutes was greater (*p* < 0.01), and of Bacteroidetes was lesser (*p* < 0.05), while the ratio of Firmicutes to Bacteroidetes was greater (*p* < 0.01) in the MY group than the LY and HY groups. The dominant genera were *Prevotella 1*, which was least in the MY group, *Ruminococcaceae_NK4A214_group*, which was least in the LY group, *Christensenellaceae_R-7_group* and *Rikenellaceae_RC9_gut_group*, which were greatest in the MY group, and *Prevotellaceae_UCG-003*, which was greatest in the LY group (*p* < 0.001). In addition, differences among groups emerged for *Unidentified, Ruminococcaceae UCG-014*, *Eubacterium coprostanoligenes group*, *Ruminococcus 2*, *Acetitomaculum*, *Lachnospiraceae XPB1014 group*, and *Veillonellaceae UCG-001* (Figure 3E; Table 6). Based on Spearman’s correlation analysis, the 10 different genera of rumen bacteria and the 10 phenotypic indicators correlation significantly (| r | > 0.6, *p* < 0.05; Figure 4). The KEGG function prediction of rumen bacterial community indicated that there were significant differences in rumen bacterial functions among groups (LDA > 2, *p* < 0.05; Figure 5).

**Table 6:** Effect of the ME:N ratio on the top 20 bacterial genera in yak rumen fluid.

Items	Group			SEM	*P*-value
	High ME:N (0.42 MJ/g)	Medium ME:N (0.36 MJ/g)	Low ME:N (0.32 MJ/g)		
*Prevotella_1*	29.3^a^	15.1^b^	21.6^c^	1.57	<0.001
*Unidentified*	15.3^b^	20.9^a^	23.1^a^	0.83	<0.001
*Ruminococcus_1*	1.08	1.08	1.00	0.15	0.970
*Saccharofermentans*	0.75	1.33	1.25	0.13	0.144
*Christensenellaceae R-7 group*	4.42^c^	8.67^a^	6.25^b^	0.44	<0.001
*Rikenellaceae RC9 gut group*	3.08^b^	7.25^a^	5.00^b^	0.48	0.001
*Ruminococcaceae NK4A214 group*	4.58^a^	5.50^a^	2.92^b^	0.33	0.002
*Ruminococcaceae UCG-014*	2.83^a^	1.92^b^	0.92^c^	0.20	<0.001
*Succiniclasticum*	5.92	5.17	4.08	0.56	0.416
*Prevotellaceae UCG-003*	1.25^b^	1.67^b^	2.75^a^	0.22	0.010
*Lachnospiraceae NK3A20 group*	1.75	2.25	1.75	0.16	0.354
*Eubacterium coprostanoligenes group*	0.92^b^	1.67^a^	2.00^a^	0.12	<0.001
*Butyrivibrio 2*	0.92	1.42	1.25	0.13	0.292
*Prevotellaceae UCG-001*	1.58	1.00	1.83	0.26	0.417
*Ruminococcus 2*	2.92^a^	0.92^b^	0.92^b^	0.32	0.011
*Acetitomaculum*	0.50^b^	1.42^a^	0.75^b^	0.12	0.002
*Lachnospiraceae XPB1014 group*	0.25^b^	1.17^a^	0.92^a^	0.11	0.001
*Veillonellaceae UCG-001*	0.42^b^	1.33^a^	1.33^a^	0.15	0.013
*Quinella*	0.00	3.00	2.42	0.67	0.156
*Succinimonas*	0.92	0.00	0.00	0.31	0.379
*Other*	21.3	17.0	17.8	1.11	0.244

a-c Different superscripts in the same row indicate significant differences(*p* ≤ 0.05).

## Discussion

Diet is the main factor shaping the rumen bacterial community and determining the utilization rate of available energy and N by rumen microorganisms (Henderson et al., 2015). The appropriate ratio of rumen substrate and N availability is not only conducive to understanding the relationship between rumen fermentation and host supply and demand, but is also vital in optimizing energy and N use efficiency and reducing environmental pollution (Ahmad et al., 2020). The results in the present study demonstrated that the ADG, DMI, blood biochemical indices, rumen fermentation parameters, and rumen bacterial structure of yaks differed when consuming diets of different ME: N ratios.

### Average daily weight gain and dry matter intake

In the present study, DMI did not differ between the ME:N ratios of 0.42 and 0.36 MJ/g, but then decreased at 0.32 MJ/g. Reports on the effect of ME:N ratio on DMI have been inconsistent. In a study by Gabler and Hendricks (2003) on Holstein heifers offered diets with an ME:N ratio between 0.54 and 0.34 MJ/g, DMI increased initially and then decreased, with a decrease in the ME:N ratio. In Chinese Holstein heifers consuming diets of ME:N ratio ranging between 0.60 and 0.44 MJ/g, DMI was not affected by the ratio (Dong et al., 2017). Some of the differences among studies could be due to differences in the composition of the diets and in the range of ME:N ratios offered the animals.

The ADG in group MY was greater than in group LY, which was consistent with the findings of Gabler and Hendricks (2003), that is, with a decrease in the ME:N (increase in proportion of N) ratio, ADG first increased and then decreased. In the study of Gabler and Hendricks (2003), Holstein heifers were offered 4 diets ranging in ME:N ratio from 0.58 to 0.34 MJ/g, and the highest ADG was achieved in the group consuming a ratio of 0.41 MJ/g. However, the effect of ME:N ratio on ADG has not been equivocal. In another study by Gabler and Hendricks (2003), Holstein heifers were offered diets differing in ME:N ratio between 0.54 and 0.34 MJ/g, and there was no difference in ADG among groups. Similarly, Dong et al. (2017) reported no difference in ADG among the three groups of Chinese Holstein heifers consuming diets of different ME:N ratios. Wittayakun et al. (2019) examined the effect of the ME:N ratio in Holstein heifers fed a pineapple waste silage-based diet with ratios between 0.65 and 0.46 MJ/g, and found no difference among groups in ADG.

### Rumen fermentation parameters

As a large anaerobic fermentation tank, the rumen provides a stable environment for the growth and proliferation of microorganisms (Flint, 1997). The microorganisms help the host digest crude fiber and crude protein and produce ammonia nitrogen (NH_3_-N), amino acids, volatile fatty acids (VFAs), and other substances, and provide energy for yaks (Shabat et al., 2016). Consequently, the pH and concentrations of NH_3_-N and VFAs are the main indicators of the status of rumen fermentation (Liang et al., 2019). The VFAs lower the rumen pH, while ammonia increases the rumen pH (Zebeli and Metzler-Zebeli, 2012). In the present study, the TVFAs and the NH_3_-N increased with a decrease in the ratio of dietary ME:N, that is, they had opposing effects on the rumen pH. Concomitantly, the rumen pH decreased, which indicated that the effect of TVAs was greater than that of NH_3_-N on the rumen pH. The TVFAs were greater in the LY than in the HY group, which was consistent with the report of Dong et al. (2017), in which the Chinese Holstein heifers increased TVFAs with an increase in the proportion of dietary N. However, Gabler and Hendricks (2003) reported no difference in TVFAs in Holstein heifers consuming diets differing in ME:N ratios.

The concentrations of ruminal NH_3_-N in the three groups in the present study were all within the optimal concentrations of 5 to 25 mg/dl (Preston and Leng, 1987). However, there was an increase in ruminal NH_3_-N concentration with a decrease in ME:N ratio, that is, with an increase in dietary N, which is consistent with results in cattle reported by Gabler and Hendricks (2003) and Dong et al. (2017). It is possible that the rate at which microorganisms degraded nitrogen-containing substances and released NH_3_-N in the rumen exceeded the rate at which microorganisms used NH_3_-N to synthesize proteins (Mahmoudi-Abyane et al., 2020). This resulted in an accumulation of NH_3_-N in rumen fluid, and more so in yaks with greater dietary N intake.

In the present study the molar proportion of ruminal acetate decreased, whereas of propionate increased with a decrease in the dietary ME:N ratio. As the ratio of dietary ME:N decreased, the dietary protein content and non-structural carbohydrates increased and of fiber decreased. Production of acetate is linked closely to fiber degradation (Wang et al., 2017), while propionate, a major substrate for gluconeogenesis, increases with a low-fiber diet (Zou et al., 2019). These relationships could explain the decrease in the molar proportions of acetate and increase in propionate with a decrease in the ME:N ratio. However, the molar concentrations of VFAs have been inconsistent when related to the ME:N ratio in cattle, with reports of no effect of the ratio on acetate, propionate, and butyrate in one study (Gabler and Hendricks 2003), but a decrease in these VFAs with an increase in the ME:N ratio in another study (Dong et al., 2017). In the present study, the ratio of acetate to propionate ranged between 3.36 and 2.60. A ratio greater than 2.2 was reported to have a positive effect on the rumen and productive performance of cattle (Russell, 1998), which would indicate that all the yaks had a well-functioning rumen, regardless of the dietary ME:N ratio (Polyorach et al., 2014).

### Blood biochemical indices

Urea is the final product of protein and amino acid metabolism, and it was reported that the concentration of serum urea was negatively correlated with nitrogen use efficiency in ruminants (Lewis, 1957). In the present study, the concentration of serum urea was highest at the highest proportion of dietary crude protein to ME, which was also reported by Gabler and Hendricks (2003) and Dong et al. (2017). This yak group also had the highest concentration of ruminal NH_3_-N, which can be explained by the strong correlation between the concentrations of ruminal NH_3_-N and serum urea (DePeters and Ferguson, 1992). The lowest serum urea concentration was in the MY group, which indicated an increased nitrogen deposition and protein synthesis (Van Bibber-Krueger et al., 2015), and was consistent with the greatest ADG in this group.

The liver is the central tissue of metabolism in animals and is involved in digestion, absorption, excretion, detoxification, material transportation, energy metabolism, and immunity (Zhang et al., 2019). Aspartate transaminase, which occurs mainly in hepatocytes, reflects the function of liver metabolism, and the serum concentration is an important indicator of the health status of the liver. When protein content in the diet exceeds requirements, it increases the liver load, and cells can be damaged (Shi et al., 2010). The permeability of cell membrane increases, aspartate transaminase in cytoplasm is released into the blood, and the concentration in serum increases. In the present study, the serum aspartate transaminase concentrations in group HY (86 U/l), group MY (85 U/l) and group LY (107 U/l) were within the normal range of 78 to 132 U/l for healthy cattle (Ingvartsen, 2006; Sun et al., 2015). However, there was a trend that the concentration in group LY was higher than in group MY, which suggests that the higher proportion of N (lower ME:N ratio) could cause liver inflammation. Concentration of serum creatine kinase is used as an indicator of gastrointestinal tract disorder (Payne and Payne, 1987). In the present study, the serum concentration of creatine kinase in group LY (363 U/l) was higher than in group MY (284 U/l) and group HY (283 U/l), and exceeded the normal range (14.7–309.8 U/l) reported for healthy water buffalo heifers (Abd Ellah et al., 2014). It is possible that group LY may have gastrointestinal irritations, but this requires further research in the expression of related genes and the structural morphology of the gastrointestinal tract. In addition, it was reported that an increase in the concentration of serum lactate dehydrogenase indicated hepatic lesions (Klein et al., 2020). In the present study, when the dietary ME:N ratio decreased from 0.42 MJ/g to 0.36 MJ/g, the concentration of serum lactate dehydrogenase was not affected, but when the ratio was further decreased to 0.32 MJ/g, the concentration of serum lactate dehydrogenase increased. Therefore, the low ratio of dietary metabolic energy to nitrogen content can potentially cause abnormal concentrations of serum biochemical-related indicators such as aspartate transaminase, creatine kinase, and lactate dehydrogenase, which could indicate liver damage.

Blood glucose is an energy source for tissues and cells in the body. In the present study, the 0.36 ME/N group had the highest serum glucose concentration and the highest ADG. It can be inferred that the increase of serum glucose concentration promoted glucose metabolism, thus improving ADG. High concentration of total protein in serum can improve the body’s metabolic level and immunity, but when the protein intake is insufficient, the serum total protein content decreases (Liu et al., 2015), which is consistent with the results of this study. With the highest ME:N ratio, and, therefore, lowest proportion of dietary N, serum concentration of total proteins decreased. However, the serum concentrations of albumin (A) and globulin (G) proteins did not differ among dietary groups in the present study and were similar to values reported for healthy cows (Alberghina et al., 2011). The A:G ratio range of 0.81 to 0.84 in the present study was within the range of 0.6 to 0.9 (Sun et al., 2015) reported for healthy cows.

### Rumen bacterial community

The rumen microbiota in ruminants is affected by dietary intake and the external environment (Weimer, 2015). In the present study, when the dietary ME:N ratio decreased from 0.42 MJ/g to 0.36 MJ/g, the α diversity index in rumen bacteria, including the observed-species, Chao1 and Shannon, increased. The difference in the results of this study may be due to the high content of corn, soybean meal, and bran in the diet. These fermentable carbohydrates were degraded rapidly, resulting in a large amount of organic substrates, and, ultimately increasing rumen bacterial abundance. However, the competition for niches among bacteria may limit the bacteria abundance, which may be the reason why the α diversity from group MY to group LY did not increase. Bacteroidetes and Firmicutes were the dominant ruminal bacterial phyla, as has been reported in buffaloes (Singh et al., 1978), steers (Myer et al., 2015), Tibetan sheep (Fan et al., 2021), and other yaks (Fan et al., 2020). Based on the bacterial functions, group MY was enriched in the TCA cycle pathway compared with groups HY and LY, which is consistent with the concentrations of serum glucose content in group MY. This may be related to the relative abundance of Firmicutes and ratio of Bacteroidetes to Firmicutes, which were greater in group MY than groups HY and LY, and they correlated positively with serum glucose concentration. Bacteroidetes can degrade carbohydrate and protein, utilize polysaccharide, and enhance fattening (Tremaroli and Bäckhed, 2012). Firmicutes are important fibrous degrading bacteria that correlates positively with an increase in body weight (Ley et al., 2006), which is consistent with the greater ADG in group MY than in groups LY and HY. Firmicutes produce large amounts of energy-rich short-chain fatty acids that are associated with digestion efficiency, and are crucial for the energy balance of ruminants (Turnbaugh et al., 2006). In addition, studies have reported that a high ratio of Firmicutes to Bacteroidetes is linked with efficient feed utilization in ruminants (Myer et al., 2015) and this could also explain, at least in part, the greater ADG in the MY than in the LY group. The general trend of increasing the relative abundance of Firmicutes and decreasing the relative abundance of Bacteroidetes suggests that the MY group was promoting fiber digestion.

At the genus level, *Prevotella* has enzymes and gene clusters necessary for fermentation and utilization of complex polysaccharides, which help to degrade protein and carbohydrate (Xue et al., 2020). This genus requires fibrous tissue or substrates after fiber degradation for optimal growth (McLoughlin et al., 2020). Therefore, the higher relative abundance of *Prevotella-1* in the HY group than the MY and LY groups, which may be due to group HY consuming the highest dietary NDF content. However, the reason for the higher relative abundance of *Prevotella-1* in group LY than group MY, although it has a lower NDF content, is uncertain and warrants further research. In the current study, there was a positive correlation between *Lachnospiraceae XPB1014 group* and average daily gain. Lachnospiraceae is important for host metabolites and maintaining a healthy ruminant intestinal environment, with some members exhibiting strong hydrolyzing activities (Louis and Flint, 2009; Sagheddu et al., 2016), which may be part of the reason for the highest ADG in the 0.36 MJ/g group. In addition, the changes of genera between groups are also reflected in *Christensenellaceae R-7 group* and *Rikenellaceae RC9 gut group*. The bacteria in the Christensenellaceae family secrete α-arabinoside enzyme, β- galactosidase, and β-glucosidase, which are related to feed efficiency (Perea et al., 2017). *Rikenellaceae RC9* plays a role in the degradation of plant derived polysaccharides (He et al., 2015; Peng et al., 2015), which is consistent with the significant positive correlations between *Christensenellaceae R-7* and the concentration of serum glucose and between *Rikenellaceae RC9 gut group* and the concentration of ruminal TVFAs. Ruminococcus is the major cellulolytic bacteria and plays an important role in fiber degradation, being rich in genes encoding cellulase and hemicellulase (Qin et al., 2022), and more abundant in ruminants fed forage-based diets (Henderson et al., 2015). With the decrease of the ratio of ME:N in the present study, the content of dietary NDF decreased, and this may be the reason for the decrease of the abundance of *Ruminococcus 2*, *Ruminococcaceae NK4A214 group*, and *Ruminococcaceae UCG-014* in the LY group. *Ruminococcaceae UCG-014* was correlated positively with the molar concentration of acetate in rumen, which is consistent with the study of Fan et al. (2021). It was reported that the relative abundance of *Acetitomaculum* was high in ruminants fed a high concentrate diet (Wang et al., 2021), and that the relative abundance increased with increasing dietary energy (Wang et al., 2019a). In the present study, the relative abundance of this genus increased with an increase in dietary energy, as expected, but then decreased with a further increase in dietary energy. The reason for this inconsistent pattern in the relative abundance of *Acetitomaculum* is uncertain and requires further research. Studies have demonstrated that there are substantial differences in rumen microbial structure and diversity between yaks and cattle (An et al., 2005; Huang et al., 2012; Wang et al., 2019b). Therefore, there are differences in the utilization of nutrient substrates between bovine species and different relationships may be formed between the host and microorganisms. Studies are needed using multi-omics technologies, including metagenomics and proteomics to determine the rumen microbial function of yaks.

## Conclusion

Average daily gain (ADG), blood biochemical indices, rumen fermentation parameters, and rumen bacterial structure differed among yaks when the actual dietary ME:N ratios were 0.42 (H), 0.36 (M), and 0.32 (L) MJ/g. The highest ADG was achieved with a ratio of 0.36 MJ/g. The relative abundance of the rumen bacteria phylum Firmicutes (F) was greater and of Bacteroidetes (B) was lesser, while the F:B ratio was greater in group M than in groups H an L. The concentration of serum glucose and the relative abundances of the bacteria genera *Christensenellaceae R-7 group* and *Rikenellaceae RC9 gut group* were greatest in this group. The 0.32 group had the highest concentration of ruminal NH_3_-N, but there were indications of liver and gastrointestinal inflammations. It was concluded that a ME:N ratio of approximately 0.36 MJ/g gave optimal results for growing yaks. However, more studies are needed to refine the optimal ME:N ratio with different feeds and on yaks of different ages, productive stages, and physiological conditions.

## Data availability statement

The datasets presented in this study can be found in online repositories. The names of the repository/repositories and accession number(s) can be found at: NCBI-PRJNA835682.

## Ethics statement

The protocol and all experimental procedures on the animals were approved by the Committee of Animal Use, Academy of Science and Veterinary Medicine, Qinghai University (Protocol QMKY202002). Written informed consent was obtained from the owners for the participation of their animals in this study.

## Author contributions

JL, JN, and YM completed the experiment. XL, DW, and AD completed the data analysis. YC, SL, and LH conceived and designed the paper. XL, YC, AD, and LH wrote and revised the article. All authors contributed to the article and approved the submitted version.

## Funding

This work was supported by The Second Tibetan Plateau Scientific Expedition and Research Program (2019QZKK0606), Key Laboratory of Plateau Grazing Animal Nutrition and Feed Science of Qinghai Province (2022-ZJ-Y17), and Top Talent project of "Kunlun Talents–High-level Innovation and Entrepreneurship Talents" in Qinghai Province.

## Conflict of interest

The authors declare that the research was conducted in the absence of any commercial or financial relationships that could be construed as a potential conflict of interest.

## Publisher’s note

All claims expressed in this article are solely those of the authors and do not necessarily represent those of their affiliated organizations, or those of the publisher, the editors and the reviewers. Any product that may be evaluated in this article, or claim that may be made by its manufacturer, is not guaranteed or endorsed by the publisher.

## Footnotes

^1^ https://www.graphpad.com/

^2^ https://www.bioincloud.tech

^3^ https://www.omicstudio.cn
